# Shared care in mental illness: A rapid review to inform implementation

**DOI:** 10.1186/1752-4458-5-31

**Published:** 2011-11-21

**Authors:** Brian J Kelly, David A Perkins, Jeffrey D Fuller, Sharon M Parker

**Affiliations:** 1 Centre for Brain and Mental Health Research, School of Medicine and Public Health, Faculty of Health University of Newcastle, University Drive, Callaghan 2308, Australia; 2 School of Nursing & Midwifery, Flinders University, Sturt Rd, Bedford Park 5024, Australia; 3 Centre for Remote Health Research, Broken Hill University Department of Rural Health, University of Sydney, Corrindah Court, Broken Hill 2880, Australia

## Abstract

**Background:**

While integrated primary healthcare for the management of depression has been well researched, appropriate models of primary care for people with severe and persistent psychotic disorders are poorly understood. In 2010 the NSW (Australia) Health Department commissioned a review of the evidence on "shared care" models of ambulatory mental health services. This focussed on critical factors in the implementation of these models in clinical practice, with a view to providing policy direction. The review excluded evidence about dementia, substance use and personality disorders.

**Methods:**

A rapid review involving a search for systematic reviews on The Cochrane Database of Systematic Reviews and Database of Abstracts of Reviews of Effects (DARE). This was followed by a search for papers published since these systematic reviews on Medline and supplemented by limited iterative searching from reference lists.

**Results:**

Shared care trials report improved mental and physical health outcomes in some clinical settings with improved social function, self management skills, service acceptability and reduced hospitalisation. Other benefits include improved access to specialist care, better engagement with and acceptability of mental health services. Limited economic evaluation shows significant set up costs, reduced patient costs and service savings often realised by other providers. Nevertheless these findings are not evident across all clinical groups. Gains require substantial cross-organisational commitment, carefully designed and consistently delivered interventions, with attention to staff selection, training and supervision. Effective models incorporated linkages across various service levels, clinical monitoring within agreed treatment protocols, improved continuity and comprehensiveness of services.

**Conclusions:**

"Shared Care" models of mental health service delivery require attention to multiple levels (from organisational to individual clinicians), and complex service re-design. Re-evaluation of the roles of specialist mental health staff is a critical requirement. As expected, no one model of "shared" care fits diverse clinical groups. On the basis of the available evidence, we recommended a local trial that examined the process of implementation of core principles of shared care within primary care and specialist mental health clinical services.

## Background

In 2008 the New South Wales (Australia) Government's Mental Health Sentinel Events Committee published its fourth report which concluded: "a definitive set of best practice standards to guide the development and implementation of shared care for mental health consumers is lacking" [1]. This Committee found cases of homicide and suicide where patients had been "under some form of shared care arrangement" but with no effective system to ensure oversight and take action where necessary. This report was referred to the New South Wales Government Department of Health and via brokerage with The Sax Institute, Sydney NSW, an expert evidence check or rapid review was commissioned.

The authors were contracted to conduct the review. This included negotiation of the research questions, presentation of preliminary scoping, draft and revised final reports. Questions and clarification were addressed via consultation involving all parties throughout the process. This form of knowledge exchange has been described as "the problem solving, policy driven model" but it might equally operate through a process of enlightenment by which evidence and ideas permeate the policy making system [2]. Rapid reviews are a new and contentious approach to knowledge transfer and exchange between researchers and policy makers. They are typically conducted in months rather than years and the focus is determined by policy-makers rather than researchers. The strength of a rapid review lies in its timeliness and responsiveness to the needs of policy makers and practitioners, but its limitations include the reliability and generalisability of the findings [3]. It has been argued that rapid reviews provide local guidance and are useful resources in the later development of a traditional systematic review [4]. To provide timely and responsive advice the search process is accelerated and the findings of existing systematic reviews emphasised, but some harder to find material in the grey literature may be missed. This may introduce biases and it has been suggested that rapid reviews should carry a warning to the effect that guidance may be revised if and when a relevant systematic review is published [5].

There is increasing recognition that improving the detection, treatment and outcomes for mental health problems requires service models that integrate mental health care within primary health care practice [6]. While much of the research to date has focussed on primary care management of depression [7], the complex needs of people with more severe and persistent mental illness has received less attention. Despite the achievements of a well-established tradition of general hospital based consultation-liaison psychiatry (comprising consultation, specialist support, collaboration and joint care) there is only limited evidence for the translation of these principles to community ambulatory and primary care [7]. Although sometimes referred to as "consultation-liaison services in primary care" [8] these models of "shared care" are often poorly defined, ranging from the simple transfer of care from one provider to another, to the involvement of one or more services in patient care, through to formalised cross-service arrangements [9].

The rapid review was undertaken to investigate the evidence underpinning "shared care" in ambulatory mental health care. The focus related mainly to adults with severe and persistent mental disorders. Evidence regarding treatment models for a broader range of conditions (e.g. dementia, substance use disorders, or personality disorder) was outside the scope of this review.

The review addressed the following questions:

1. Does "Shared Care" improve clinical outcomes for people with mental illness?

2. If so, what are the proposed ingredients of an effective "Shared Care" program?

The latter required hypothesising a proposed set of such ingredients based on a synthesis of the literature.

## Methods

The research team comprised a psychiatrist with research, clinical and teaching responsibilities, a nursing academic with research and clinical experience in mental health services, a health systems researcher specialising in mental health and primary health care and an information scientist with mental health nursing experience. The group had recently completed a 12 month narrative review of effective service linkages in primary mental health care [10] were familiar with the literature and had established working arrangements. They also had many years of experience with the NSW health care system and brought a strong understanding of service and political context.

Shared care covers a broad spectrum of collaborative treatment arrangements and there is no standard definition in the literature [11,12]. For the purposes of this rapid review, we developed the following definition based on the published literature:

*A structured system for achieving integration of care across multiple autonomous providers and services *[13]*with both primary and secondary care practitioners contributing to elements of a patient's overall package of care *[9]. *Shared care involves some agreement about the shared activities and levels of responsibility for each provider and appropriate communication processes to support this integration. A shared care arrangement may involve any combination of government, non-government or private sector providers.*

As a rapid review, the initial step was to identify systematic reviews and examine their relevance. This was followed by a search for papers published subsequent to these systematic reviews as well as iterative searching from reference lists for any outstanding published works. The search for systematic reviews was conducted in the Cochrane Database of Systematic Reviews and Database of Abstracts of Reviews of Effects (DARE) (2004-2010). A comprehensive search strategy was developed and applied to Medline (Additional file 1). One person (SP) assessed each title and abstract against the inclusion and exclusion criteria (Additional file 2) and consulted with a second author where there was any uncertainty. Focussed data extraction was undertaken and extracted material discussed and explored during project meetings.

We found six systematic reviews of shared or collaborative care that included people with a mental disorder [10,11,14-17], several covered depression in some depth [10,15-19]. We then reviewed 271 citations from the black search of which 23 dealt with mental health conditions other than adult depression. We included 12 studies of severe or persistent mental illness (21 citations). Two addressed bi-polar disorder [20,21], nine severe or chronic mental illness [22-29], and one first episode psychosis [30].

## Results

### Does shared care work?

The benefits of shared care models are summarised in Table 1. The highest quality evidence (level 4) [31] comes from US and UK studies of the treatment of mental disorders in primary care, such as depression and anxiety (often in the context of coexisting physical conditions such as diabetes, cancer or cardiovascular disease). These trials mainly recruited those aged 60 or over [32,33].

**Table 1:** Evidence for the benefits of shared care

Benefit	Supporting evidence
**Access and Equity**	• Improved access by reducing barriers to availability of integrated care with primary care or improved access to specialist care (Druss 2001, Harrison-Read 2002, PRISM-E, van Orden 2009).
	• Improved access to outpatient services, rehab services, and an increase the number of people receiving follow-up, case management and review of their medication (Byng 2004, Gilmer 2010, Rosenheck 2003)
	• Increased ability to target high priority groups through tailored programs linked with relevant services (e.g. cultural groups, age-based services, homelessness) (Asanow 2009, Gilmer 2010, Rosenheck 2003)
	• Reduced impact of perceived stigma on help seeking for mental health problems (PRISM-E, Gavin 2008)
**Acceptability and stigma**	• Reduced unmet need for treatment (PRISM-E (73% of participants))
	• Improved cultural appropriateness of service (IMPACT, PRISM-E)
**Comprehensiveness**	• Increased efficacy of pharmacological/psychological treatments in primary care. Treatment course is generally predictable and with good step up/step down algorithms (IMPACT, PROSPECT,CALM, Bower 2006, Gilbody 2006))
	• Capacity to match intensity of intervention to patient need (stepped care) (IMPACT, PROSPECT, CALM)
	• Improved capacity of generalist services to meet full range of patient needs (IMPACT, PRISM-E)
	• Improved communication between levels of care (primary and specialist) (Craven 2006, PRISM-E)
	• Improved capacity to address mental health aspects of physical illness and chronic disease (Byng 2004, Druss 2001, Gilmer 2010, Rosenheck 2003, PRISM-E, IMPACT)
	• Improved skill of generalist health worker in mental health care (Fuller 2009)
	• Considers client preference in the choice of care delivered (IMPACT, CALM)
	• Promotes engagement in care (e.g. via link worker) (Byng 2004, Oxman 2003)
**Continuity**	• Single point of contact to review care progress and needs (e.g. through primary care) (Druss 2001, PRISM-E)
**Outcomes: clinical, functional. social**	• Evidence of improved clinical outcomes (psychiatric) (Bauer 2006, Bertelsen 2008, Bower 2006, Fuller 2009, Gilbody 2006, Simon 2006, PRISM-E (6 mths only), IMPACT, PROSPECT, RESPECT-D, CALM)
	• Evidence of improved clinical outcomes (physical and medical) (Druss 2001, IMPACT)
	• Evidence of reduced hospitalisation for mental health problem (Bauer 2009)
	• Evidence of improved social functioning and/or quality of life (Bauer 2009, Gilmer 2010, Rosenheck 2003, IMPACT)
	• Greater satisfaction with care (Asarnow 2009 (6 months), Bauer 2009, Gilmer 2010, Rosenheck 2003, PRISM-E, IMPACT, RESPECT-D)
	• Increased ability of consumers to manage their own care (IMPACT)
	• Reduced or equal client costs (Bauer 2006, Bower 2006, Druss 2001, Katon 2002, van Orden 2009)
**Cost**^**1**^	• Increased health care costs of initial set-up (first 12 months) balanced against cost savings in following year (Katon 2002, Katon 2006, Simon 2007)
	• Reduced in-patient costs (Bauer 2009, Byng 2004, Druss 2001)
	• Cost-offset effects on non-mental health-related ambulatory care services (IMPACT)
	• Reduction in costs to other systems (e.g. justice) (Gilmer 2010, Rosenheck 2003)

^1 ^Costs generally have been assessed during the intervention period only - up to 2 years follow up in large depression trials

#### Depression and other common mental disorders

Smith [11] undertook a detailed meta-analysis examining 20 studies of shared care for chronic disease management, six of which examined shared care for depressive disorders and 3 persistent mental illness, the remainder investigating a range of shared care interventions for chronic physical conditions. The most consistent finding was in prescribing and drug treatment adherence in favour of shared care interventions. The six studies of shared care for depression alone reported evidence of benefits in rates of recovery or remission [33-38]. Nevertheless, Smith drew the sobering conclusion that overall "consistent evidence for the effectiveness of shared care is lacking for most of the outcomes studied". The authors go on to conclude that effectiveness may be greater for clinical subgroups, highlighting findings among patients with depressive disorders particularly.

While most studies to date have focussed on depression either alone or in combination with chronic physical illness, a recent RCT investigated a flexible treatment delivery model for collaborative primary care management of people with multiple anxiety disorders (CALM) [39]. This study demonstrated significant improvements in anxiety and depressive symptoms, functional disability and in measures of quality care.

A meta-analysis of interventions targeting improved communication strategies between primary care providers and specialists (representing a model of "shared care" for the purposes of our review) by Foy [14] found moderate overall effect sizes for a range of chronic conditions, including mental disorders (18/38 studies) with similar effect sizes across conditions. This review also found that effect size increased when analysis was restricted to studies of depression. This supports the results of meta-analyses of depression trials [15,17] that have reported positive clinical outcomes, increased use and effective treatment with antidepressants and sustainable benefits for up to 5 years.

Two large US trials on collaborative care for depression (IMPACT and PRISM-E) also reported results for subgroups from ethnic minorities [40,41] with both reporting similar results to the general patient population studied.

#### Severe and persistent mental disorders

There were fewer published papers about the benefits of "shared care" for severe and persistent mental disorders (e.g. schizophrenia) and very little work about service links outside the health sector with welfare, housing, education, or employment.

Despite a high need for health and social services for people with such persistent psychotic disorders, Fuller [10] found only 16 of 119 reviewed studies examined such services. Of the RCTs, three reported some clinical benefit including improved mental and physical function [42] improved physical function [23] and reduced relapse rate [22].RCTs by Lester and Warner [26,43] of patient-held records found no clinical or service use benefits, while a cohort study by McDonough [44] reported no clinical or service use benefit from a GP-Community Health Team shared care register. However, an RCT by van Orden [29] found no clinical benefit from an on-site mental health worker in primary care, but reported decreased waiting time to see a mental health professional and decreased mean number of referrals for specialised mental health appointments. Other studies of co-located services have reported improved communication among providers, and increased referral to mental health services [45,46].

Studies of Assertive Community Treatment (ACT) [20,47,48], found no significant clinical outcomes but improved social function, quality of life and satisfaction, decreased hospitalisation and improved engagement with services. A trial by Druss [23] of veterans with severe mental illness also reported service improvements. Those receiving integrated medical and mental health care were compared with those receiving usual care in a general medicine clinic. After one year, veterans assigned to the integrated care clinic reported significantly improved access and quality of care (e.g. fewer problems in access to mental health services, greater attention to consumer preferences, courtesy, and coordination). The largest effect was in continuity of care.

Two studies of supported housing and mental health follow up using ACT principles for people with persistent and severe mental illness reported improved quality of life, better physical health, higher satisfaction and improvements in housing stability and perhaps because of this, improved treatment engagement [24,27]

We found no evidence of programs reporting adverse clinical outcomes.

### What are the necessary components of effective shared care services?

On the basis of the existing studies, a major focus of this review was the inductive process to propose or hypothesise a set of "core ingredients" that comprise effective shared care, providing a base to inform future service development projects.

It was evident that models of shared care varied in complexity reflecting the target population, the structure and organisation of the health services in which they were located, and other local factors [49]. Shared care covered a broad spectrum of collaborative treatment arrangements, where provider involvement fluctuated depending on consumer need and so there was no single model to fit every community or situation [12]. Regrettably, while studies have demonstrated some benefits from these multi-component interventions across patient outcomes, service use and some costs, there is less evidence from such studies regarding the *process *of service change required to achieve these outcomes.

In general, effective collaborations were found to use multi-component strategies that reflected the following: i) the chronic and complex nature of many mental health problems, ii) the need to improve access to and engagement in treatments (e.g. through co-location of general and mental health services to lessen stigma), iii) the need to improve treatment adherence and continuation through care coordination, facilitation and monitoring [50] and iv) strategies that applied a clear set of core ingredients to promote service collaboration but with capacity for flexibility to local needs and context [51]. Without differentiating between the type of mental illness, Fuller [10] identified that most trials with positive clinical, service use or economic outcomes employed multiple linkage strategies that include some "direct collaborative activity", "guideline initiated care" and "a communication system". For the purpose of this review we have tabulated five of the major international studies on shared care for depression and anxiety (Table 2) and twelve for severe and persistent mental illness (Table 3 and Table 4) in an attempt to examine the common components of such interventions across these studies. Common components across both the depression/anxiety and severe and persistent mental illness studies are case management and care coordination roles (chiefly with mental health expertise), clinical supervision, follow up and use of standard outcome measures. Treatment algorithms were most evident in the depression/anxiety studies but not in the studies of persistent mental illness. The use of specific strategies targeting enhanced communication between providers is particularly evident in the persistent mental illness studies.

**Table 2:** The core components of effective shared care models for depression and anxiety

	PRISM-E	MPACT I	PROSPECT	RESPECT-D	CALM
Process of care	Integrated mental health service	Care manager (nurses or psychologists trained for the study	Care manager (depression care manager)	Care manager (background in PC or MH nursing)	Flexible treatment delivery model (preferred treatment) Anxiety Care Specialist (ACS) to deliver web based CBT program
Screening	yes	yes	yes	no	yes
Treatment algorithm	no	yes	yes	no	yes
Formal stepped care	no	yes	yes	no	yes
Care management location	na	on-site	on-site	off-site (centrally located)	on-site
Patient education/self management	variable	yes	yes	yes	yes
Case management	yes	yes	yes	yes	yes
Care management to patient contact	na	face-to-face; telephone	face-to-face	telephone	face-to-face
Psychiatric supervision	na	face-to-face; telephone	face-to-face	telephone	telephone/email
Care management counselling	na	PST-PC	IPT	supportive	supportive
Psychological supervision	na	telephone	face-to-face	na	unclear
MH specialty treatment location	on-site	on-site	on-site	off-site	on-site
Geriatrician supervision	no	Liaison/PC provider	no	no	no
Standardised follow up	yes	yes	yes	yes	yes
Standardised outcome measure	yes	yes	yes	yes	yes

**Source**: Oxman -The American Journal of Geriatric Psychiatry 2003; 11, 5- (Table 2, Page 509)

Butler - AHRQ Publication No. 09-E003. Rockville, MD. Agency for Healthcare Research and Quality. 2008 (Table 4, Page 46)

Roy-Byrne - Delivery of Evidence-Based Treatment for Multiple Anxiety Disorders in Primary Care. A Randomized Controlled Trial JAMA 2010;303,19.

**Table 3:** The core components of shared care models for severe and persistent mental disorders

	COPERATIVE STUDIES PROGRAM 430 Bauer 2009	Simon 2006	OPUS Bertelson 2008	MENTAL HEALTH LINK Byng 2004	Druss 2001	Gilmer 2010
Process of care	Specialty mental health team (.5 fulltime-equivalent (FTE) nurse and a .25 FTE psychiatrist)	Nurse care managers with at least 5 years of clinical psychiatric experience	Assertive Community Treatment, family treatment and social skills training	Facilitation based QI programme designed to improve communication between general practice and community mental health and improve systems of care within general practice (including roles of link worker and psychiatrist)	Integrated primary care and mental health clinic	Full service partnerships and subsidised housing and full fidelity Assertive Community Treatment by team- based services with a focus on rehabilitation and recovery

Condition	Bipolar disorder and associated co-morbidities including: substance use disorders, anxiety disorders, any current psychiatric co-morbidity and active medical co-morbidity requiring treatment	Bipolar spectrum disorder diagnosed during previous 12 months (bipolar disorder type I or type II, schizoaffective disorder, or cyclothymia).	1^st ^episode psychosis	Long term mental illness - chronic psychosis, and disabling neuroses	SMI & homeless; co-morbid drug and alcohol abuse.	SMI (schizophrenia, bipolar disorder, or major depression)

Length of follow up	3 years	2 years	2 and 5 years	1 year	1 year	2 years

Screening	yes	yes	yes	yes	no	unclear

Additional training for staff	yes	yes	yes - staffed trained to deliver early intervention program	yes - training of research facilitators	no	no

Treatment algorithm	yes - used to promote identification and treatment by outlining medications to use without sequencing individual agents	yes	no	no	no	unclear

Formal stepped care	no	yes	no - team assessed as to when patients were ready for a specific treatment modality	no	no	unclear

Enhanced communication between health providers	no	yes- contact tracking, structured assessment, and standardised feedback reports to providers	unclear	yes - formal communication guidelines around referral, discharge and professional roles and patient management	yes - e-mail, telephone, and face-to-face discussion	unclear

Care management location	outpatient clinic	behavioural health clinics	primary care office or in patient's home or other places in the community	general practice	primary care clinic and mental health clinic adjoining	community

Patient education/self management	yes	yes	yes - focus on problem solving and development of skills to cope with illness	no	yes	no

Case management	yes	yes	yes- team based	yes	yes	yes

Specialist supervision	yes	yes - weekly	yes	yes	yes	yes

Care coordination	yes - scheduling appointments and follow-up for missed appointments, and with mental health and medical-surgical providers	yes	yes - across team and social services and other involved institutions	yes	yes - scheduling appointments and follow-up of missed appointments between the two clinics	unclear

Follow up provided to patient	yes	yes	yes	yes	yes	yes

Crisis support	yes	yes	yes - crisis plan developed with each patient. Patients given out of hours contact number for response the following day	unclear	unclear	yes - 24/7

Standardised outcome measure	yes	yes	yes	yes	yes	yes

**Table 4:** The core components of shared care models for severe and/or persistent mental disorders (contd)

	Harrison-Read 2002	Lester 2003	ACCESS Rosenheck 2002	Rosenheck 2003	van Orden 2009	Warner 2000
Process of care	Enhanced Community Management/Assertive Community Treatment	Patient-held record	Integration of service systems with outreach and case management	Housing + Intensive Case Management	Collaborative care involving access to a mental health worker in primary care	Shared care record

Condition	"Heavy users" of psychiatric services	Schizophrenia	SMI and associated co-morbidities + homelessness	Psychiatric and/or substance abuse disorders	Mental disorder (not described)	Long term mental illness- psychosis, personality disorder or other condition requiring long term supervision

Length of follow-up	2 years	1 year	5 years	1 year	1 year	1 year

Screening	no	no	yes	unclear	yes	Patients selected at hospital discharge

Additional training for staff	unclear	yes - in use of the record	yes - inter-agency	yes - with written materials		no

Treatment algorithm	unclear	no	unclear	no		no

Formal stepped care	unclear	no	unclear	no		no

Enhanced communication between health providers	yes	yes - shared care record and flagging of patient records in both general practice and specialist settings	yes	yes - inter-agency agreement	yes	yes - shared care record linked to other communication processes

Care management location	community	general practice and community	community	community	general practice/primary care	general practice and community

Patient education/self management	unclear	no	unclear	no	yes - CBT	no -instruction on use of the booklet only

Case management	yes	unclear	yes	yes	?	unclear

Specialist supervision	yes	unclear	yes	unclear	yes	unclear

Care coordination	yes	unclear	yes	yes	no - referral only	unclear

Follow up provided to patient	unclear	unclear	yes	unclear	unclear	unclear

Crisis support	no	unclear	unclear	yes	unclear	unclear

Standardised outcome measure	yes	yes	yes	yes	yes	yes

Based on a synthesis of these findings, the following discussion proposes specific elements of effective shared care services while acknowledging this complexity and the overlap of these elements.

#### Clarity of Intervention

Studies demonstrating improved outcomes for depression and anxiety using "shared care" in primary care have usually been well articulated clinical treatment models within an established theoretical framework such as Problem-Solving Therapy [33].The evidence suggests that successful shared care requires a clear clinical focus tailored to the needs of particular conditions or patient subpopulations, specific selection of patients, coherent treatment models and agreed strategies to monitor treatment progress. These include, step-wise escalation and de-escalation of treatment intensity (type, frequency and duration) or urgency of response as needed and with mechanisms to ensure the maintenance of the necessary skills to deliver interventions [50,52,53] such as expert clinician supervision. Some studies also utilised patient education and self-management resources within stepped care intervention models. Some studies explicitly included patient treatment preferences [39]. This is an important feature since intervention studies need effective treatment options that are acceptable to consumers for broader implementation [54].

In contrast, studies of persistent and severe mental disorders such as schizophrenia have often entailed service brokerage and linkage interventions with less emphasis on particular therapeutic strategies or clearly articulated service models. Rather, they focus on the process and organisational aspects of care and ensuring engagement and communication across service sectors, such as housing, employment, hospital and community-based care [20]. Studies in these populations also suggest that the benefits of specialist and primary care jointly working together are most evident when there is face to face contact with experienced mental health workers for case discussion than just case register or proactive recall strategies [44,51].

#### Staff attributes and skill

Fuller [18] found that the attributes and skills of staff were important and linkages were enhanced when key staff had experience in both mental health and primary care [55-57], and when clinical staff had a flexible work style that helped them to fit into a team [55,58,59].The majority of studies targeting patients with persistent and severe mental illness, incorporated roles for identified mental health professionals, working within primary care to implement the shared care intervention with a range of tasks including care coordination, case review and liaison activities with primary care. Referred to in some instances as "link workers" [60], such roles were aimed to build "linkages" between primary care and specialist mental health services. Byng [60] found that the level of experience and flexibility of such specialist mental health link workers was important, particularly to achieve the benefits of their participation in face to face consultation for patients in "crisis". When staff doubted the value of a collaborative strategy, such as a formalised referral method, then this has resulted in low uptake of the strategy [63,64].

While some studies suggest outcomes are better when care coordination is provided by mental health trained practitioners [17] others find that these skills can be developed by non-specialist health care providers if supported by training, regular case supervision and mentorship. These elements allow for direct patient consultation by specialists if needed [61,62] or for face to face consultation between primary care and specialist clinicians when needed [21].

While the need to increase the supply of mental health professionals is widely accepted, in some instances evidence suggests that efficient and effective use of specialists can support integrated delivery of care within primary health care sector. Improving the confidence, capacity and engagement of primary care clinicians in mental health care does however require attention to their support, training and supervision and to the attitudinal barriers to mental health service provision [63]. Jointly managed and funded collaborative care arrangements may help to address these barriers. This can be achieved while retaining the capacity to respond to the needs of the most complex patient [6].

#### Purposively designed care delivery system

Butler [64] noted that most trials of collaborative care built on the Chronic Care Model [65] include self-management support, a purposively designed care delivery system, decision support tools and a clinical information system.It is interesting to note the findings from a "Realistic Evaluation" of the UK-based program established by [51], which reveals the importance of contextual factors in tailoring the intervention to local needs, based on a mixture of fixed and flexible components that enabled a shared care agreement that reflected the local context. It is also interesting to note that electronic data register systems were perceived as less important in achieving the goals of this shared care intervention. Findings from this study suggest that so-called "catalyzing" functions (i.e. the integration of a mental health link worker into the primary care team, and facilitated planning) and "doing" (e.g. face to face discussion of patients and provision of advice when required,) were more effective than interventions based on improving information technology [60].

#### Clarity of roles

Role clarity is an important component of collaborative development and sustainability [23,66]. Yaffe [67] found that in 40% of cases primary care and mental health clinicians disagreed about the responsibility for and purpose of a referral (i.e. whether to assess or to treat). In the IMPACT study on depression, team and worker role clarification was used to identify safe practice boundaries [56].

#### Leadership and governance

Butler noted that overcoming local organisational and cultural barriers requires strong leadership to champion change [5].*S*hared governance arrangements between primary care and specialist services were found to support faithful application of evidence-based shared care models [60,68,69]. Such clinical governance was needed to promote (1) clear, agreed lines of clinical accountability, (2) clinically appropriate interventions linked with clear methods to ensure appropriate clinical supervision of staff [17,70], and (3) appropriate evaluation of the "shared care" model [17,39,50,52]. Formal service agreements were found to build organisational support for "shared care". In several trials this was an important means to establish a mandate, change leadership and provide resources for integration [55,57,71-73].

#### Funding

The ACCESS trial found that mental health and human services that received direct funding and technical support achieved better integration [74], while Butler [64] described financial and organisational barriers to collaborative service arrangements including: the lack of reimbursement for activities such as care planning with other providers, and the restrictive rules imposed when combining funds from different sources; difficulties employing staff with the skills for new roles crossing mental health and primary care; and the costs of collaboration.

In most studies the research provided additional staffing and organisational infrastructure that was "grafted" onto existing services, with some service re-orientation. However, some small effectiveness trials did provide a more robust "bridge" to routine practice by utilising existing staff and resources [22,53] such as identification of mental health link workers, with researchers providing facilitation, tools and limited funding to support project implementation [21].

#### Physical infrastructure

Physical infrastructure was also important. The provision of co-located accommodation was an enabler of collaboration, when mental health care managers were located in the primary care clinic enabling optimum team visibility and interaction [56], but a barrier when clinic accommodation was inadequate [75]. Interestingly low uptake of quality improvement (QI) programs designed to increase collaboration between primary care and other specialist mental health and community mental health services has been attributed to a lack of face to face contact between primary care and mental health specialists [65].

#### Feedback about outcomes

In the IMPACT study [56,71]"*the most important factor, cited in four of the five sites, was the ability to document positive client outcomes from the research study*". Mechanisms to feedback evidence of outcomes to team staff seems to have been a key contributor to developing and sustaining service collaboration in this study, however the value of this process was not reported more widely in the other reviewed studies.

## Discussion

"Shared Care" between specialist and generalist services has been a major focus of mental health service reforms. The purpose of this rapid review was to examine published evidence about models of shared care in mental health with specific attention to the core ingredients and processes necessary to support effective shared care models. The available evidence suggests that models of "shared care" between primary health services and specialist mental health service can lead to improved clinical outcomes in some clinical groups, such as depression and anxiety disorders. There is a small amount of evidence that shared care can provide better outcomes for people with psychoses and related disorders, such as reduced relapse rates. The complexity of the systems of care necessary for people with these disorders, may explain the limited evidence for shared care in this setting.

The literature to date has used inconsistent definition of shared care; hence it is not surprising that it is difficult to distil core elements of effective care from existing studies. Furthermore the majority of the evidence is drawn from studies addressing depression and anxiety. There is less evidence regarding shared care in severe and persistent mental disorders and we cannot readily assume that models developed for the former will work with the latter.

On the basis of this rapid review we deduced a number of core ingredients of effective shared care models. These comprise provision of collaborative care in a coherent, evidence-based clinical management framework with:

i) A systematic approach to the engagement of primary and specialist services towards the common goal of improved mental health care

ii) A coherent treatment model relating to the target condition/s or patient population,

iii) An agreed clinical pathway and monitoring of patient outcomes with the provision of case review by specialist personnel when needed

iv) Attention to staffing requirements and the provision of clinical supervision to support skill development and maintenance of treatment model [50,53].

v) A well-established clinical governance framework.

In studies of shared care for people with persistent and severe mental disorders, a common element was the provision a care coordinator acting as a link between primary care and specialist mental health services.

A broader issue is the challenge of the translation of evidence into clinical practice. The gap between evidence-based care and clinical practice is widely acknowledged. While many studies demonstrate the benefits of multi-component interventions for patient outcomes, service use and cost, there is less evidence about the successful implementation of such models into routine care. Changing clinician behaviour (within both primary care and specialist sectors) and maintaining systems that support and promote such changes is believed to be key to the implementation of new models of care [76]. The "voltage loss" over time of such interventions is a clear risk [50].

There are a number of important limitations to this review. A number of compromises to standard methods for systematic review were made. The shortened timescale did not permit activities to be undertaken sequentially and so activities overlapped and material was analysed as it became available. We were only able to examine a small selection of local grey literature. Particular importance was placed on published systematic reviews and the search focussed on key questions that were not addressed in those reviews, chiefly shared care for those with severe and persistent mental disorders. Despite these limitations we attempted to ensure high standards of replicability and transparency through defined inclusion and exclusion criteria [4] as detailed in Additional file 2.

It was not possible to undertake meta-analytic evaluation of the strength of evidence as studies investigated complex interventions, with varying levels of attention to the methodological and statistical issues [77]. Second, while papers often focused on specific desirable clinical outcomes, a detailed description of the service implementation process was often poorly reported. This is a key requirement for the translation of such findings to practice.

## Conclusions

From many angles, it is imperative to improve the links between primary care and specialist mental health services. When joint primary and specialist level collaborative care models have been evaluated using RCT designs, a range of clinical and service benefits are reported, particularly in the primary care management of depression and anxiety disorders.

The available evidence provides only limited support for shared care models for the treatment of persistent psychotic disorders. While a few robust and innovative studies have been undertaken with this population further studies are needed, including research that encompasses diverse geographic and health service contexts.

Some core components of shared care warrant further targeted research (e.g. the specific functions and roles of mental health linkage workers). Demonstration projects with detailed and innovative evaluative designs are needed to understand better these core elements and the processes that underpin effective shared care, particularly their capacity to be translated to routine clinical practice.

The strategies proposed in this review pose substantial implementation challenges for any health service, yet are critical to ensuring that shared care services are better structured to achieve sustained improvements in mental health care.

## Competing interests

The authors declare that they have no competing interests.

## Authors' contributions

BK led the preparation of the manuscript, participated in review of studies, collation and interpretation of findings. DP contributed to the manuscript, led the project team, and participated in review of studies, collation and interpretation of findings. JF contributed to the manuscript, participated in the review of studies, collation and interpretation of findings. SP designed the search strategy and conducted the literature searches, was responsible for data extraction and data management. She contributed to the interpretation of the findings and the drafting of the manuscript.

All of the listed authors have read and approved the final manuscript.

## Supplementary Material

Additional file 1 **Search Strategy for Medline**. Detail of comprehensive search strategy for this review. Click here for file

Additional file 2 **Exclusion and Inclusion Criteria**. Detail of specific criteria for papers included in this search. Click here for file
