# Supplementary material for: Shared care in mental illness: A rapid review to inform implementation
Source: Int J Ment Health Syst. 2011 Nov 21;5:31. doi: 10.1186/1752-4458-5-31 (PMC3235059; doi:10.1186/1752-4458-5-31)
Supplement: Additional file 1 — Search Strategy for Medline. Detail of comprehensive search strategy for this review. [file 1752-4458-5-31-S1.PDF]

Additional file 1: Search Strategy for Medline

The following search strategy was applied to Medline (9th July 2010)

1 shar$3 care.tw,hw. (655)
2 shared mental health care.mp (20)
3 (integrated adj3 care).tw,hw. (8030)
4 (collaborative adj3 care).tw,hw. (935)
5 exp Interprofessional Relations/ (43640)
6 enhanced communication.mp. (151)
7 exp Interdisciplinary Communication/ (5119)
8 exp Cooperative Behavior/ (17673)
9 consultation liaison.mp. (987)
10 exp "Delivery of Health Care, Integrated"/ (6246)
11 exp Health Care Costs/ (35608)
12 exp clinical governance/ (89)
13 exp "Costs and Cost Analysis"/ (150697)
14 exp risk management/ (141480)
15 1 or 2 or 3 or 4 or 5 or 6 or 7 or 8 or 9 or 10 or 11 or 12 or 13 or 14 (351502)
16 exp Mental Disorders/ (773637)
17 suicide risk.mp. (1603)
18 exp "Diagnosis, Dual (Psychiatry)"/ (2331)
19 exp Homeless Persons/ (5170)
20 15 or 16 or 17 or 18 or 19 (777268)
21 exp Dementia/ (92956)
22 20 not 21 (684555)
23 exp primary health care/ (59951)
24 exp Community Mental Health Services/ (15090)
25 exp housing/ (21171)
26 exp social welfare/ (59842)
27 exp Community Health Services/ (414178)
28 23 or 24 or 25 or 26 or 27 (527124)
29 14 and 22 and 28 (4136)
30 quasi-experimental.mp. (3096)
31 randomized controlled trial.pt. (293554)
32 randomised controlled trial.mp. (6014)
33 controlled clinical trial.pt. (81810)
34 controlled clinical trial.mp. (87384)
35 (time adj series).tw. (9243)
36 (pretest or post test).mp. (8125)
37 29 or 30 or 31 or 32 or 33 or 34 or 35 or 36 (390641)
38 29 and 37 (417)
39 limit 38 to (english language and humans and yr="1999 -Current" and "all adult (19 plus years)") (250)
