# Supplementary material for: Shared care in mental illness: A rapid review to inform implementation
Source: Int J Ment Health Syst. 2011 Nov 21;5:31. doi: 10.1186/1752-4458-5-31 (PMC3235059; doi:10.1186/1752-4458-5-31)
Supplement: Additional file 2 — Exclusion and Inclusion Criteria. Detail of specific criteria for papers included in this search. [file 1752-4458-5-31-S2.PDF]

Additional file 2: Inclusion and exclusion criteria

Participants and setting/s

Inclusion:

- Adults (18years plus) with a mental disorder with or without a co-morbid drug and/or alcohol condition and/or a chronic physical condition or disability
- All disciplines involved in the delivery of mental health care including GPs, psychiatrists, mental health nurses/workers, psychologists, welfare workers, social workers or counsellors etc.
- At least two services from either the primary health, community (including non-government organisations), or tertiary sectors providing mental health care to consumers

Exclusion:

- Participants receiving care in a correctional facility
- Participants with a primary diagnosis of dementia
- Participants with psychiatric symptoms related to intellectual disability
- Active members of military organisations whose care is provided by that organisation

Shared care Interventions

Inclusion:
We will include only studies where there is evidence that providers in the study had an explicit agreement about their shared responsibility and levels of responsibility via one or more of the following:

1. Explicit process of communication and enhanced information exchange over and above routine discharge and referral notices including:

- Those involving a primary care and specialist provider where the ongoing management of patients is discussed and planned. This includes face to face meetings and meetings conducted using telephone and videoconferencing
- Activities between members of at least two organisations involved in the delivery of mental health care where the management of patients is discussed and planned. This includes face to face meetings and meetings conducted using telephone and videoconferencing
- Shared-care records (patient-held) and computer-assisted shared care and electronic mail, where an agreed data set is collected in both primary and specialty-care settings, and is circulated between sectors or available to both parties at a central location. This system could also include centrally coordinated computerised registration and recall of patients.

2. Explicit process for care management (including a designated coordinator to link services) providing each of the parties involved contributes to the planning of care and there is agreement or agreed protocols for the delivery of care by each party.

Exclusion:

- Disease management taking place entirely within general practice including specialist clinics in general practice
- Private arrangements between a GP and a specialist provider i.e. does not meet the definition of a structured and formally documented system of care
- Transfer of care from one service to another
- Professional educational interventions or research initiatives where no specified, structured clinical care is delivered to patients
